# Acute benzo[a]pyrene exposure induced oxidative stress, neurotoxicity and epigenetic change in blood clam *Tegillarca granosa*

**DOI:** 10.1038/s41598-021-98354-5

**Published:** 2021-09-21

**Authors:** Baoying Guo, Dan Feng, Zhongtian Xu, Pengzhi Qi, Xiaojun Yan

**Affiliations:** National Engineering Research Center of Marine Facilities Aquaculture, Marine Science and Technology College, Zhejiang Ocean University, Zhoushan, 316004 Zhejiang China

**Keywords:** Environmental sciences, Gene expression analysis

## Abstract

The blood clam (*Tegillarca granosa*) is being developed into a model bivalve mollusc for assessing and monitoring marine pollution on the offshore seabed. However, the information on the response of blood clam to PAHs, an organic pollutant usually deposited in submarine sediment, remains limited. Herein, we employed multiple biomarkers, including histological changes, oxidative stress, neurotoxicity and global DNA methylation, to investigate the effects of 10 and 100 μg/L Bap exposure on the blood clams under laboratory conditions, as well as the potential mechanisms. Acute Bap exposure can induce significant morphological abnormalities in gills as shown through hematoxylin–eosin (H.E) staining, providing an intuitive understanding on the effects of Bap on the structural organization of the blood clams. Meanwhile, the oxidative stress was significantly elevated as manifested by the increase of antioxidants activities of superoxide dismutase (SOD), catalase (CAT), peroxidase (POD) and glutathione-*s*-transferase (GST), lipid peroxidation (LPO) level and 8-hydroxy-2′-deoxyguanosine (8-OHdG) content. The neurotoxicity was also strengthened by Bap toxicity manifested as inhibited acetylcholinesterase (AChE) and choline acetyltransferase (ChAT) activities. In addition, the global DNA methylation level was investigated, and a significant DNA hypomethylation was observed in Bap exposed the blood clam. The correlation analysis showed that the global DNA methylation was negatively correlated with antioxidants (SOD, CAT and POD) activities, but positively correlated choline enzymes (AChE and ChAT) activities. These results collectively suggested that acute Bap exposure can cause damage in gills structures in the blood clam possibly by generating oxidative stress and neurotoxicity, and the global DNA methylation was inhibited to increase the transcriptional expression level of antioxidants genes and consequently elevate antioxidants activities against Bap toxicity. These results are hoped to shed some new light on the study of ecotoxicology effect of PAHs on marine bivalves.

## Introduction

Polycyclic aromatic hydrocarbons (PAHs), a class of persistent organic pollutants (POPs), widely and small amounts present in water environment. However, with the rapid development of urbanization and industrialization, the content of PAHs in water environment continues to increase by atmospheric deposition and industrial wastewater discharge^1^. In aquatic systems, PAHs are easily absorbed in particulate matter then deposited in the sediment to negatively impact benthic organisms, and part of PAHs will be released from the sediment and enter other aquatic organisms to influence the whole food web and ecosystem^2^. Benzo[a]pyrene (Bap), a model PAHs, is generally known as a kind of mutagenic, teratogenic, carcinogenic and ubiquitous environmental contaminant^3^. Previous studies have shown Bap pollution in coastal areas of China is relatively serious, and the concentration of Bap in the most polluted areas can be as high as 4.799 μg/L in seawater and 2.640 μg/g in dry weight^4^. The blood clam (*Tegillarca granosa*) widely distributed along the coast of Indo-Pacific region, represents an economically significant bivalve species in China marine fisheries^5^. *Tegillarca granosa* lives in the intertidal mudflats in shallow waters, and are sessile or sedentary, filter seeding, these characteristics confers them more susceptible to Bap pollution^6^. Considering the above factors, it is of great significance to investigate the toxic effects of Bap on *T. granosa*, and the results will provide some helpful information for the fishery resource protection of *T. granosa.*

A prior study has shown that once Bap get into the aquatic organisms, a step-wise mechanism called phase I and II metabolism are triggered to convert the Bap into hydrophilic metabolites^7^, then excreted from the body. In essence, phase I metabolism is an oxidation–reduction reaction that produce reactive oxygen species (ROS) and toxic intermediate metabolite. ROS is the main cause of oxidative stress. Excess ROS can lead to oxidative damages, which mainly represented by lipid peroxidation (LPO), DNA damage, protein oxidation, etc.^8^. LPO is often used as a kind of biomarker of oxidative stress and damage. Malondialdehyde (MDA) is an important product in the process of lipid peroxidation, typically, the content of MDA is measured to judge the degree of LPO^9^. Among all studied biomarkers for oxidative stress, DNA damage is recognized as a strong discrimination factor to evaluate the toxicity effect of various contaminants, and 8-hydroxy-2′-deoxyguanosine (8-OHdG) is recognized as one of the cardinal manifestations of DNA damage^10,11^. 8-OHdG has been demonstrated to be an effective biomarker to assess Bap induced oxidative DNA damage in a number of bivalves such as common mussels *Mytilus galloprovincialis*^12^, green-lipped mussels *Perna viridis*^13^ and scallop *Chlamys farreri*^14^. The phase I metabolism of Bap is mediated by antioxidative system. The superoxide dismutase (SOD) is involved in the catalyzing of superoxide anion leading to the formation of hydrogen peroxide, which will further be converted to water and oxygen by catalase (CAT) and peroxidase (POD). In phase II metabolism, glutathione-*s*-transferase (GST) is the central component. It participates in Bap metabolism and detoxification by catalyzing the intermediate metabolite in phase I metabolism to increase its hydrophobicity^15^.

In comparison with its induced oxidative stress, the neurotoxicity of Bap has received considerably less attention^16^. Similar to most lipophilic compounds, Bap can easily cross the blood–brain barrier and thereby gains direct access to the central nervous system^17^. It has been shown that environmental exposure to Bap correlates with impaired learning and memory in adults^18^, and poor neurodevelopment in children^19^. An animal experiment on the mechanism of neurotoxicity indicates that the acute neurobehavioral toxicity induced by Bap may occur through oxidative stress by inhibiting the brain antioxidant scavenging system^20^. The cholinergic system has shown important regulatory functions in learning and memory behavior as confirmed by a number of autopsies^21,22^. Choline is acetylated by the enzyme choline acetyltransferase (ChAT) to yield acetylcholine (ACh)^23^, thereafter ACh is hydrolyzed into choline and acetic acid by acetylcholinesterase (AChE) to ensure effective transfer at the cholinergic nerve^24^. ACh is a central neurotransmitter widely spread along the synaptic cleft and participates in the regulation of neuronal activity in the hippocampus and neocortex. Unfortunately, due to its rapid rate of hydrolysis, ACh is very unstable and difficult to determine. Therefore, the monitoring of the cholinergic system is usually executed by observing the activities of AChE and ChAT^25^. In bivalves, AChE is often selected as the neurotoxicity biomarker to investigate the effects of environmental pollutants. Some field studies showed significant repression on AChE enzymic activities in *Mytilus sp.* collected from polluted site with respect to control, and generally attributed these fluctuations to PAHs^26–30^. Similarly, laboratory studies also shown that acute Bap exposure significantly depressed the AChE activity in gills or digestive gland of *M. galloprovincialis*^31,32^. In comparison with the high yields of AChE as neurotoxicity biomarker, ChAT is relatively less applied as a neurotoxicity biomarker. Maisano et al.^29^ found a depletion in ChAT in gills of *M. galloprovincialis* caged at Augusta (a petrochemical polluted site in Italy “Augusta-Melilli-Priol” region) for 60 days. Conversely, in gills of the same mussel species caged for 30 days at Priolo, a site within the “Augusta-Melilli-Priolo” industrial area and thus suffering as well as from petrochemical pollution, Cappello et al.^26^ reported an enhancement of ChAT.

Epigenetic refers to the direct and indirect modifications to DNA that can impact gene expression without altering the underlying nucleotide sequence^33^. Various mechanisms including DNA methylation, chromatin remodeling, non-coding RNAs and histone modification have the potential to encode epigenetic information. These epigenetic endpoints are sensitive to environmental stimuli such as exposure to contaminants, physiological stress, parental behavior, and nutritional deficits, and therefore suitable to develop into a promising pollution biomonitoring tool^34–36^. DNA methylation is the most well studied and common epigenetic mechanism, where methyl groups are transferred to cytosine bases of CpG dinucleotides to form 5-methylcytosine^37^. The Bap or Bap metabolite can impair the activity of DNA methyltransferase (DNMT), in turn leading to a decrease in DNA 5-methylcytosine content in cell lines, has been awared since early 1980s^38–40^. In recent years, studies in human cohorts exposed to PAHs have shown the further relationships between exposure and potential altered methylation status of offspring^41–44^. In zebrafish embryos, waterborne Bap exposure significantly decreased global DNA methylation, further suggesting that aberrant DNA methylation could be involved in the Bap-induced toxicity^45,46^. The initial research on DNA methylation in bivalves focused on the relationship between gene function and methylation pattern. In *Crassostrea gigas*, genes with housekeeping functions are more methylated than genes involved in inducible functions^47–49^. The emerging studies in recent years have shown that DNA methylation play an important regulatory role in development and organism-environment interaction in bivalves^50–53^. However, there is no research on the functional role of DNA methylation in the Bap exposure response of bivalves. During exposure to Bap, it is unclear whether the DNA methylation of bivalves has the same function as mammals.

Herein, we firstly investigated the histological changes in gills between control and Bap exposed *T. granosa*, to obtain an intuitive knowledge on the effects of Bap toxicity on *T. granosa*. Next, we employed multiple biomarkers to analyze the effects, and we assess the oxidative stress (indicated by antioxidants (SOD, CAT, POD and GST), lipid peroxidation (MDA) and oxidative DNA damage (8-OHdG)), neurotoxicity (indicated by AChE and ChAT) and global DNA methylation fluctuation induced by acute Bap exposure in gills. These results are hoped to shed some new light on the study of ecotoxicology effect of PAHs on marine bivalves.

## Results

### Histopathology observation

**Figure 1:**
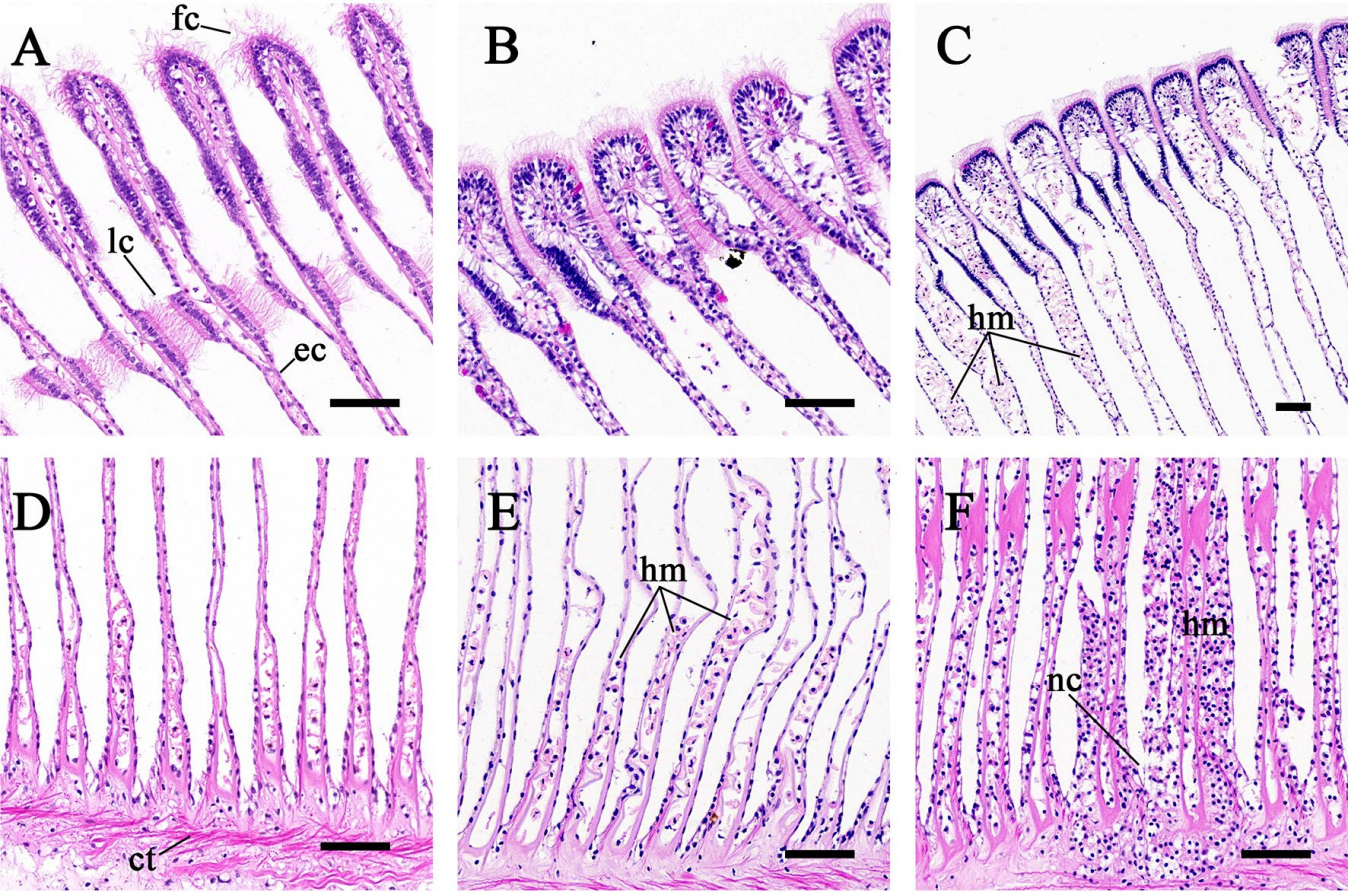
Representative histopathological micrographs of the gills of blood clams. (**A** and **D**) control; (**B** and **E**) exposed to 10 μg/L Bap; (**C** and **F**) exposed to 100 μg/L Bap. *fc* Front cilia, *lc* lateral cilia, *ec* epithelium cell, *hm* hemocyte, *nc* necrosis, *ct* connective tissue. Scale bar: 50 μm.

As shown in Fig. 1A and D, the gills of control group exhibited well-preserved structure with barely histopathological changes. Normal gills revealed a typical fine structure of the bivalve ctenidium. All gill filaments consisted of a single layer of epithelial cells and had two types of cilia (frontal cilia and lateral cilia). They attached to supporting cartilage and arranged regularly. When the blood clams were exposed to 10 μg/L Bap, morphological changes of gill filaments accompanied by hemocyte infiltration were obviously observed (Fig. 1B, E). The top of gill filaments was swollen and connected together by lateral cilia. Detachment of some epithelial cells led to terminal rupture. The base of gill filaments was significantly distorted and deformed. In the 100 μg/L Bap treatment individuals, the gill filaments were filled with hemocytes, which responsible for engorgement. The breakage and necrosis became more severe (Fig. 1C, F).

### Effects of Bap on antioxidants activities

**Figure 2:**
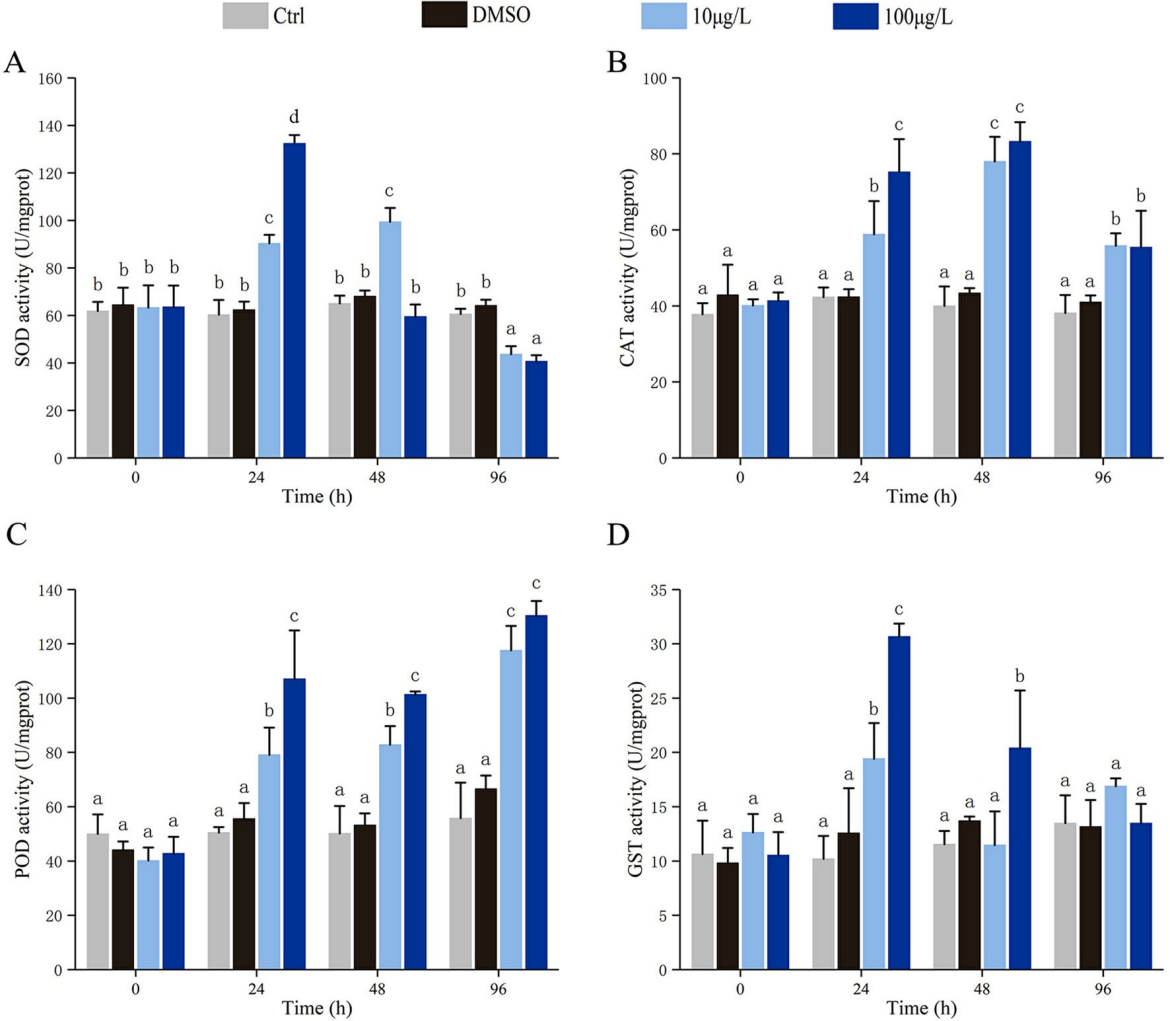
Effects of acute Bap exposure on activities of SOD (**A**), CAT (**B**), POD (**C**) and GST (**D**) in gills of blood clams. The vertical bars represent the mean ± SD (n = 3). Different letters indicate significant differences (*p* < 0.05).

As shown in Fig. 2, the activities of antioxidative enzymes including SOD, CAT, POD and GST all respond to Bap exposure in gills. SOD, CAT and GST activities roughly showed a trend of first rising and then falling (Fig. 2A, B, D). In 10 µg/L Bap group, SOD activity increased gradually and reached the peak level at 48 hpe, followed by a sharp decrease to a level lower than control at 96 hpe (Fig. 2A). Once exposed to 100 µg/L Bap, SOD activity was sharply elevated, and showed a significant increase at 24 hpe compared to control. After that, SOD activity rapidly reduced to the same level as control at 48 hpe, whereas an inhibited level than control at 96 hpe (Fig. 2A). CAT activity in both treatment groups gradually increased and peaked at 48 hpe, then declined rapidly, but was still higher than control at 96 hpe (Fig. 2B). GST activity rose rapidly to the highest level at 24 hpe and then gradually declined, but was still higher than control at the end of the experiment (Fig. 2D). However, POD activity in both treatment groups showed a trend of gradual increase and reached the peak level at 96 hpe (Fig. 2C).

### Effects of Bap on LPO level

**Figure 3:**
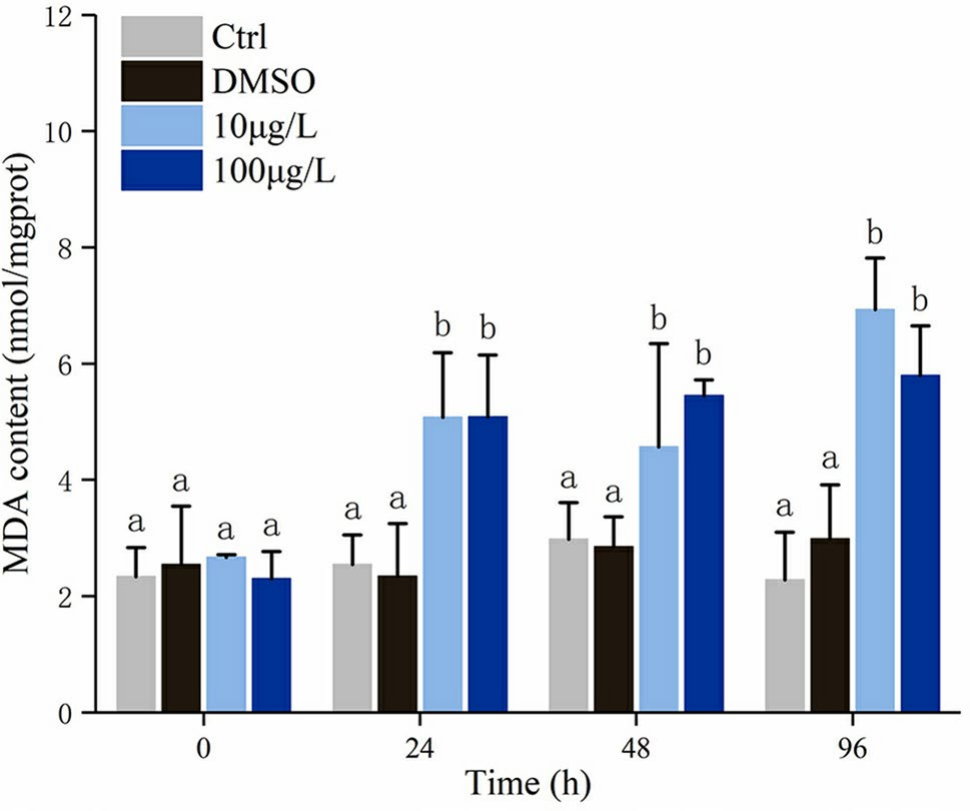
MDA content in gills of blood clams exposed to 10 and 100 μg/L Bap. Values represent mean ± SD (n = 3). Different letters indicate significant differences (*p* < 0.05).

LPO level was manifested by the MDA content. Upon exposed to Bap, MDA content in gills significantly increased, and showed a time-dependent manner (Fig. 3). The highest level of MDA content was detected at 96 hpe, with a 3.04- and 2.54-fold increases in 10 and 100 µg/L Bap groups, respectively (Fig. 3).

### Oxidative DNA damage

**Figure 4:**
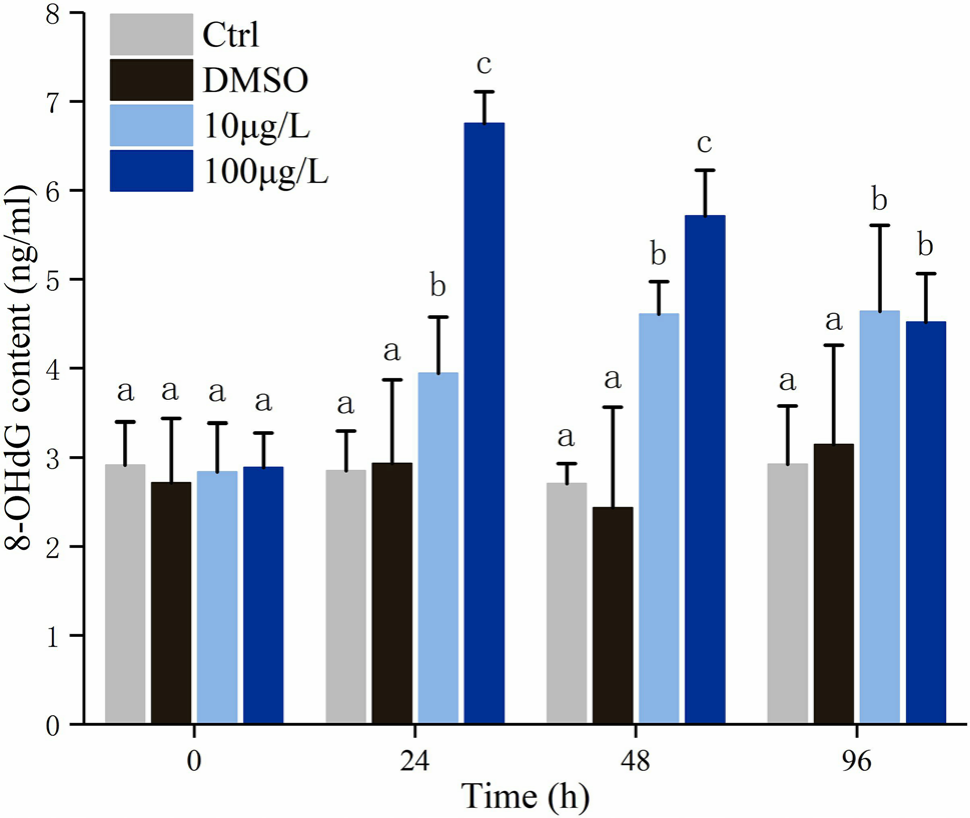
Levels of 8-OHdG in gills of Bap exposed blood clams. The vertical bars represent the mean ± SD (n = 3). Different letters indicate significant differences (*p* < 0.05).

Oxidative DNA damage induced by Bap was manifested by the 8-OHdG content. As shown in Fig. 4, Bap exposure significantly elevated the level of 8-OHdG. When exposed to 100 µg/L Bap, the level of 8-OHdG sharply increased and peaked at 24 hpe, after then gradually decreased as the exposure duration elapsed, but was still significantly higher than control at the end of the experiment (Fig. 4). However, 8-OHdG content in 10 µg/L Bap exposed gills shown a trend of gradually increase, and the highest level was detected at 96 hpe (Fig. 4).

### Neurotoxicity assessment

**Figure 5:**
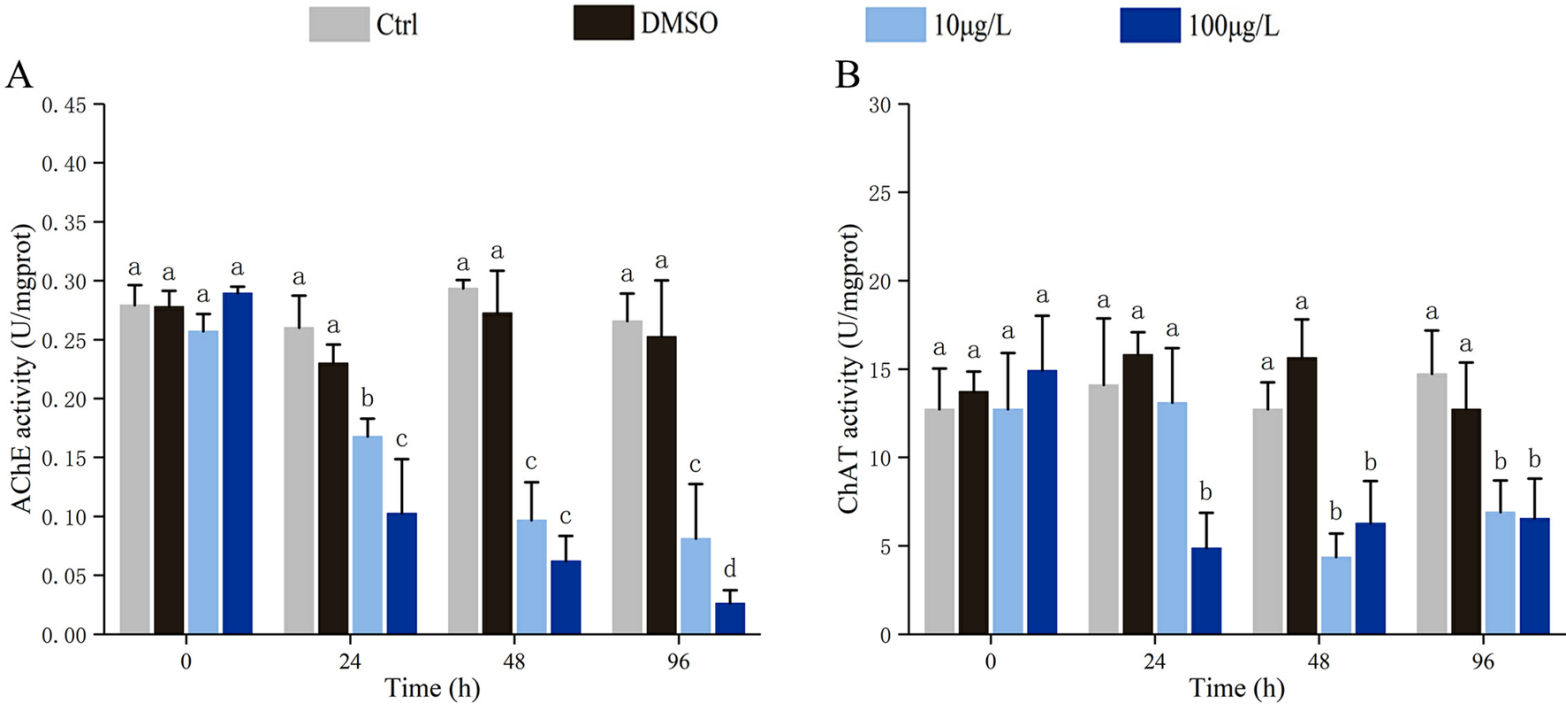
The AChE (**A**) and ChAT (**B**) levels were significantly inhibited in gills of blood clams upon exposure to Bap at the concentration of 10 and 100 μg/L. The vertical bars represent the mean ± SD (n = 3). Different letters indicate significant differences (*p* < 0.05).

AChE and ChAT levels were used to assess the neurotoxicity induced by Bap in blood clams’ gills. As shown in Fig. 5, a significant inhibition of AChE and ChAT activities was observed in Bap exposed gills. Once exposed to Bap, AChE activity rapidly declined and showed a time-dependent trend, with the lowest AChE activity detected at 96 phe (Fig. 5A). ChAT activity was also significantly inhibited by Bap, but there was no significant difference between different time points (Fig. 5B).

### Global DNA methylation

**Figure 6:**
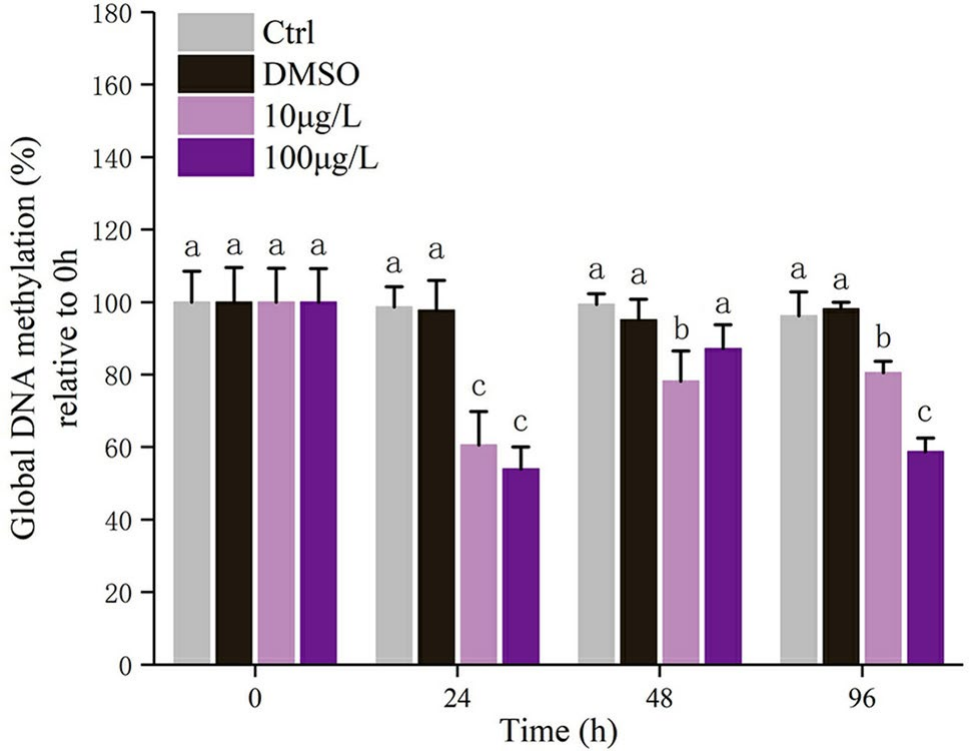
Effects of acute Bap exposure on global DNA methylation levels in gills of blood clams. The vertical bars represent the mean ± SD (n = 3). Different letters indicate significant differences (*p* < 0.05).

As shown in Fig. 6, the DNA methylation level decreased significantly in gills of blood clams exposed to Bap. After exposure to Bap, the DNA methylation level decreased rapidly at 24 hpe, and transiently increased at 48 hpe but was still significantly lower than the control, and dropped significantly at 96 hpe (Fig. 6).

### Correlation analysis

**Figure 7:**
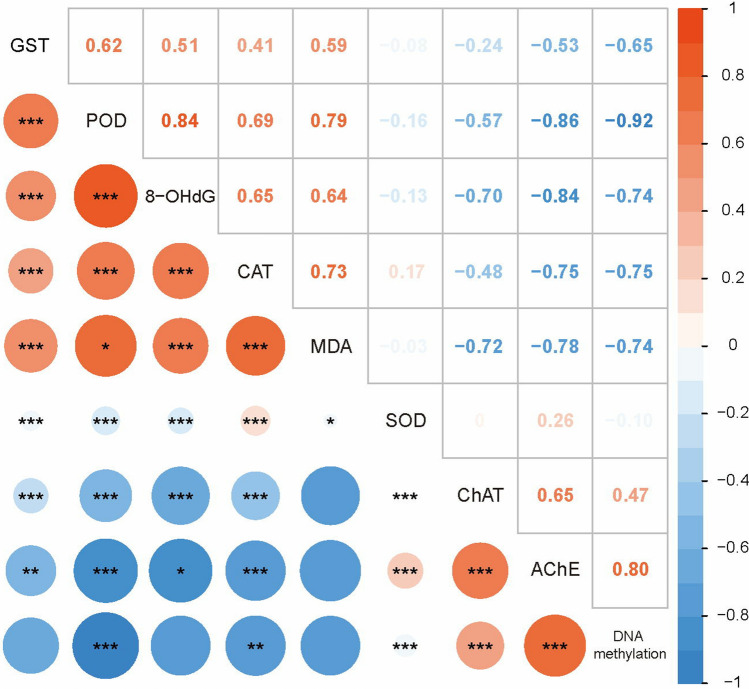
Spearman rank correlation results for all indicators. The circle size is proportional to the correlation value (Spearman rank correlation coefficient), which represents correlation level. Red represents positive correlation and blue represents negative correlation. *p* values: *p ≤ 0.05, **p ≤ 0.01, ***p ≤ 0.001.

Correlations between all indicators after Bap exposure were summarized in Fig. 7. In this Figure, the global DNA methylation level was shown to strongly positively correlated with AChE activity, and strongly negatively correlated with the activity of POD and CAT at a significant level (*p* < 0.01). Meanwhile, the results indicate significant (*p* < 0.01) but weak correlation between the global DNA methylation level and ChAT activity (positive correlation) or SOD activity (negative correlation). Similarly, the 8-OHdG content presented distinct correlation levels with all antioxidant or detoxification enzymes activities except SOD (positive correlation) and the two neurotoxicity biomarkers (negative correlation) at a significant level (*p* < 0.05). On the other hand, the activity of antioxidant or detoxification enzymes presented an overall positive correlation between each other, while presented an overall negative correlation between the activity of AChE and ChAT.

## Discussion

The blood clam is an economically and ecologically significant bivalve species along China coastal waters. The demersal lifestyle makes them more susceptible to Bap pollution which are prone to be deposited in the sediment. However, there are very few studies on the effects of Bap on blood clams. Su et al.^6^ investigated the immunotoxicity induced by Bap under ocean acidification conditions, which is the only searchable study on the Bap toxicity on blood clams to our knowledge. Here, we performed acute Bap exposure on blood clams, and then assessed the effects of Bap toxicity: histological changes, oxidative stress, neurotoxicity and global DNA methylation.

As filter-feeding organisms, the gill tissues of molluscs are exposed to the external environment and function as the main entrance for waterborne toxicants, exerting the first environment-tissue barrier against contaminants^54^. Aiming to provide an intuitive understanding on the effects of Bap on the gills of blood clams, we investigated the histological changes in gills by means of H.E staining. Under Bap exposure of present nominal concentration, some significant morphological abnormalities including gill filaments denuded of cilia, hemocyte infiltration and consequent breakage and necrosis, were observed. Meanwhile, the damage degree of gills seemed like Bap dose dependent. Similarly, in mussels *M. galloprovincialis* from the petrochemical polluted site, severe morphological alterations were also observed in gills^29^. Speciale et al.^55^ exerted experimental exposure of *M. galloprovincialis* to 0.5 and 1 mg/L Bap, and found that high levels of Bap exposure resulted in loss of structural integrity characterized by detachment and degeneration of epithelial cells, sloughing of epithelium and denuded of cilia. Additionally, the severity of the damage was dependent on Bap concentration and exposure duration^55^. These findings collectively indicated that petrochemical pollutants containing Bap could induce high oxidative stress to bivalve molluscs and lead to morphological alterations in organ tissues for instance in gills. Due to the important physiological significance of mussel gills in feeding efficiency and gas exchange^56^, the destruction of this structural organization may affect the functional integrity of the gills^57^.

Oxidative stress refers to an increase in intracellular ROS levels that leads to damage to lipids, proteins and DNA^8^. It has been demonstrated as a primary Bap-mediated mechanism of toxicity in the molluscs^58^. Here, we investigated the Bap induced oxidative stress as manifested by the change of antioxidative enzyme activities, LPO level and oxidative DNA damage in blood clam gills. SOD-CAT is recognized as the first line antioxidative defence against excessive ROS, and POD plays a significant role in following converting for hydrogen peroxide to hydrogen peroxide^59^. The significant elevation of SOD, CAT and POD enzymic activities after Bap exposure implied the production of oxidative stress in *T. granosa* gills. Followed, phase II metabolism was triggered to detoxify the Bap which was suggested by increased GST level. Similar results were also detected in other bivalve molluscs. When green-lipped mussel *Perna viridis* exposed to 0.3 and 3 µg/L Bap for 18 days, SOD and GST capacity increased in the gills. However, CAT activity remained unchanged^60^. In scallop *Chlamys farreri*, SOD, CAT and GST enzymic activities in gills increased in short time at 0.5 and 1.0 µg/L concentration of Bap, but significantly depressed at 10.0 and 50.0 µg/L concentration^61^. In mussels *M. galloprovincialis*, 10 µg/L Bap exposure for 7 days elevated the enzymic activities of CAT and GST enzymes in gills, but no change SOD activity^62^. When clam *Ruditapes philippinarum* was exposed to 0.01 µg/L Bap for 21 days, the activities of SOD and GST were significant up-regulated at early stage of exposure (3–6 days), and then declined to the control level when the experiment terminated^63^. After 6 days exposure of Bap at 56 µg/L, SOD activity in *M. coruscus* gills increased, but no significant induction of CAT activity^64^. Collectively, these results provided a clear viewpoint that Bap could induce severe oxidative stress in bivalve molluscs. Notably, the fluctuation trend of specific enzyme response to Bap exposure was not consistent in different studies. In our opinion, the concentration and duration of Bap exposure, the sampling points, the sensitivity of various molluscs to Bap might lead to the fluctuation discrepancy of specific enzyme in different studies.

ROS generated from redox cycling progress of Bap cause macromolecules damage^65^, and the MDA and 8-OHdG are extensively recognized as effective biomarkers for macromolecules damage, and the former for LPO level, the latter for oxidative DNA damage. The MDA content exhibited a gradual increase with the exposure duration, suggesting that Bap exposure can time-dependently elevate the LPO level of blood clams, consequently leading to cytotoxicity. Similar results were also observed in scallop *Chlamys farreri* that MDA increased with the exposure time and there was a positive correlation between the MDA content and the concentration of Bap^61^. The presence of 8-OHdG during DNA replication is known to mislead DNA templates at both modified and adjacent bases in vitro^66^. When mussels *M. galloprovincialis* was subjected to a wide dose-range of waterborne Bap, a significantly increased 8-OHdG level was detected in gills whereas no significant dose–response relationships were observed between 8-OHdG and Bap^12^. However, when the same species was exposed to food-borne Bap, there was no apparent fluctuation in 8-OHdG in gills^31^. These researchers supposed that different exposure manners of Bap, viz. feed supply and waterborne, leaded to different 8-OHdG levels in the same mussel gills. In a field study to examine the efficiency and efficacy of biomarkers for environmental carcinogens monitoring, 8-OHdG levels in *Perna viridis* mussels between control and polluted sites showed no significant differences^13^. In addition, over the complete 30-day exposure period, no significant correlations between 8-OHdG and organic contaminant concentrations in tissues were observed. However, at some sampling points in specific polluted sites, positive correlation was observed between Bap and 8-OHdG, whereas negative correlation was observed at another sampling point in another polluted site. Given these results, the researchers suggested that 8-OHdG was unlikely to be a good biomarker in field studies^13^. The present results were likely to indicate a time-dependent increase of 8-OHdG in response to 10 µg/L Bap exposure. This seemed at odds with previous researches, perhaps depending on the intensity and the duration of the stress applied on the one hand and on the susceptibility of the exposed living species on the other hand^67^. Notably, in in vitro study of the effects of metabolism enzymes on Bap-induced DNA damage in the scallop *Chlamys farreri*, 8-OHdG content increased significantly by inhibiting GST and SOD^14^. Conversely, in the present study, the 8-OHdG content showed a significantly positive correlation with all examined antioxidants except SOD. The underlying mechanism for these discrepancies was uncertain. Despite of these, the present results demonstrated that Bap exposure could induce an oxidative DNA damage in blood clam gills, and this induction was time-dependent in Bap treatment at low concentration. Notably, in 100 µg/L Bap group, the 8-OHdG in gills was found to reach the peak at 24 hpe, followed by a gradually decrease with the time elapse. This was discrepancy with observed in low concentration Bap group. We speculated that Bap exposure at high concentration might transiently induce the severe DNA damage, along with cellular structural destruction, resulting in insufficient DNA synthesis to neutralize 8-OHdG in subsequent periods.

Neurotoxicity has been largely overlooked in the risk assessments of Bap^16^. In a few studies using F344 rats as the model animals to investigate the neurotoxic effects for Bap, apparent neurotoxic symptoms such as physiological and autonomic activity dysfunction, weakened reaction to stimuli were exhibited after exposure to Bap^20^. ACh is a cholinergic neurotransmitter which plays a central role in regulation of the neuronal activity, generally used as an important index that reflects cholinergic nerve function^25^. However, ACh is very unstable and difficult to determine due to its rapid rate of hydrolysis, and therefore the functioning of the cholinergic system is usually observed through the activities of ChAT and AChE, which the former is responsible for ACh synthesis through catalyzed reaction of choline with acetoacetyl-CoA, whereas the latter exerts the hydrolysis of ACh into choline and acetic acid^23^. The measurement of AChE inhibition in marine organisms has been widely used as an indicator of neurotoxicity caused by environmental contamination. AChE activity has been demonstrated to be strongly inhibited by organophosphate and carbamate pesticides, and also by metals and surfactant agents^68–72^, causing dysfunction of the central nervous system. In mussels *M. galloprovincialis*, AChE activity was significantly reduced after exposure to Bap^31,32^. Similarly, in the present study, a remarkable decrease of AChE activity was also found in blood clams with exposure to Bap. The reduction of AChE activity could constrain the hydrolysis of the ACh^73^, lead to neuro-transmission perturbation and cell apoptosis, and consequently weakening the neural system and causing the gill tissue in an obtuse state to external stimulus^74^. These findings provided the direct evidence that Bap produce neurotoxicity in molluscs just as it has done in mammals, and AChE index could be used as an indicator to monitor Bap contamination. However, the underlying mechanism for inhibited AChE activity by Bap was unknown, perhaps irreversible or reversible binding to the catalytic site of the enzyme and potentiation of cholinergic effects provides certain contribution^30^.

ChAT is the rate-limiting enzyme of ACh synthesis, which can be used as an indirect evaluation index of acetylcholine release level^25^. Even so, there were relatively few studies on ChAT as an indicator of neurotoxicity. In Alzheimer's disease, a typical neurological disease of humans, the activity of ChAT was found to decrease dramatically in some specific brain regions in patients, and deemed to be closely related to the degree of dementia^75^. In gills of mussels *M. galloprovincialis* caged for 60 days at Augusta, a site within the “Augusta-Melilli-Priolo” industrial area and thus suffering from severe petrochemical pollution, Maisano et al.^29^ reported an inhibition of both AChE and ChAT, indicating that cholinergic function was severely compromised, and this may result in impairment of the ciliary function and filtering activity of gills. Similarly, a statistically significant inhibition of AChE and ChAT in gills was found in mussels *M. galloprovincialis* collected from Lake Faro (Sicily, Italy), a natural confined brackish environment subjected to PAHs contamination^27^. Bap is a model PAHs, which is a major component of petrochemical pollutants, therefore these results indirectly suggested that ChAT in molluscs was also sensitive to Bap pollution. The current detection of significant inhibition of ChAT activity in the gills of Bap exposed blood clams further strengthens this view. Conversely, in gills of mussels caged for 30 days at Priolo, another site suffering as well as from petrochemical pollution, an inhibition of AChE coupled with an enhancement of ChAT was detected, suggesting that paracrine signaling mediated adaptive compensatory responses were possibly triggered to recover a regular physiological function of gills^26^.

DNA methylation has been demonstrated to be involved in the stress response to Bap exposure in mammals and model organisms. Smith and Hansch^76^ found that 5′-methylated cytosine at CpG islands was the preferred binding site for Bap in cigarette smoke, indicating that this site may be a target for lung cancer caused by Bap. In zebrafish *Danio rerio*, Bap exposure leaded to reduced global and gene specific DNA methylation^45,46^. Reduced DNA methylation of specific genes can increase the transcriptional expression level, which in turn involved in multiple pathogenic mechanisms including growth defects, embryo malformation and etc. Regrettably, so far, there have been no studies on the involvement of DNA methylation in response to Bap exposure in molluscs, and therefore no parallel data for us to compare the present result. However, the detected remarkably decrease of global DNA methylation here is consistent with previous findings in zebrafish, suggesting at least that DNA methylation in blood clams is involved in the stress response to Bap toxicity just as its correspondent epigenetic mechanism done in vertebrates. Notably, the majority of methylation in vertebrates occurs in non-coding intergenic regions of the DNA^77^. Hypomethylation of these regions, specifically gene promoters, typically results in prompting gene expression by opening transcriptional machinery^78^. Bap decreases the global DNA methylation level in zebrafish, leading to the induction of genes related to environmental stress by turning on the switch in gene promoters, and then participates in the fight against Bap toxicity. However, the mechanism maybe different in blood clams. In contrary to vertebrates, methylation of invertebrate DNA primarily exists in coding intragenic regions, suggesting a potentially different role for DNA methylation in invertebrates^47^. The global DNA methylation reduction by Bap in blood clams might allow for fine-tuning of transcripts and of responses through transient methylation^48^. After exposure to copper, the global level of hydroxymethylcytosine in *C. gigas*, a most studied molluscan species in epigenetic scenario, was found to be reduced^79^. Similarly, a decrease on global DNA methylation level was also detected in *Physa acuta* with the exposure of vinclozolin and prednisolone, respectively^80,81^. On the contrary, cadmium exposure was found to induces global cytosine (and possibly hydroxycytosine) hypermethylation in *C. aspersus*^82^. Despite of the not strictly consistent results, these findings at least suggest that DNA methylation could play a significant role in defense against oxidative stress induced by multiple xenobiotics in molluscs. The correlation analysis between global DNA methylation and antioxidants provided an additional evidence, that the global DNA methylation was significantly negatively correlated with antioxidants activities in gills of Bap exposed *T. granosa*. We speculated that hypomethylation upon Bap exposure could turn on the switch in gene promoters to trigger these antioxidants transcriptional expression, and consequently elevated the levels of antioxidative activities to confront Bap toxicity. In addition, other mechanisms including exon skipping and transcription start sites substitution caused by DNA methylation may also contribute to the variation in gene expression and resultantly improve response to Bap toxicity^48^. There was a positive correlation observed between global DNA methylation and choline enzymes activities, the underlying mechanism for this was uncertain. Considering that inhibition of AChE activity was associated with neurotoxicity, the current results at least suggested that Bap induced DNA hypomethylation indirectly correlated with neurotoxicity.

Herein, we performed a laboratory study to investigate the effects of Bap exposure on *T. granosa.* Multiple biomarkers, including histological changes, oxidative stress, neurotoxicity and global DNA methylation, were employed in the present study. Acute Bap exposure can induce significant morphological abnormalities in gills by generating oxidative stress and neurotoxicity. The global DNA methylation was inhibited, and possible correlated with elevated antioxidants activities against Bap toxicity. Meanwhile, Bap induced DNA hypomethylation may also be indirectly associated with neurotoxicity.

## Materials and methods

### Chemicals and reagents

The Bap and dimethyl sulfoxide (DMSO) were purchased from SIGMA-ALADRICH (Shanghai, China). The enzyme activity assay kit for SOD, POD, CAT, GST, AChE, ChAT enzymes and the enzyme linked immunosorbent assay (ELISA) kit of 8-OHdG were purchased from Nanjing Jiancheng Bioengineering Institute (Nanjing, China). The global DNA methylation was investigated using the MethyFlash Global DNA Methylation (5-mC) ELISA Easy kit (EPIGENTEK, Farmingdale, NY, USA). All other reagents were obtained from SOLARBIO (Beijing, China) unless other mentioned.

### Animals

Healthy blood clam adults (length: 29.87 ± 3.23 mm, weight: 9.43 ± 1.32 g) were purchased from Donghe market (Zhoushan, Zhejiang Province, China). Before experiment, the blood clams were acclimated in plastic tanks filled with artificial sterile seawater (ASW) at 23 ± 1 °C temperature, 25.0 ± 0.5‰ salinity, and 8.1 ± 0.2 pH value for one week, and approximately 8 animals for liter of ASW. During acclimation, *Platymonas subcordiformis* was used to fed the blood clam twice daily at a rate of ~ 5% dry tissue weight^83^, and the ASW was renewed daily to remove excess food and excreta.

### Bap exposure and tissue sample collection

DMSO was used as the vehicle of Bap and added to ASW to reach a final concentration of 0.01% (v/v). After acclimation, a 96 h acute Bap exposure experiment was performed. A total of 240 blood clams were randomly divided into control group (ASW), solvent control group (DMSO), and two Bap exposure (10 and 100 μg/L) groups. The chosen Bap concentration in this experiment was according to Su et al.^6^ and Tang et al.^84^ and represented at par with the low and high concentration, respectively. The experiment contained triple replicates and 20 individuals in each replicate. During experiment, all conditions were the same with domestication, but no feeding. The gills of three clams were randomly sampled at 0, 24, 48 and 96 h post exposure (hpe) and pooled together as one sample to minimize variations between individuals.

The concentrations of Bap in water samples were determined by GC–MS method according to Di et al.^85^. Briefly, an AGILENT 5975 GC–MS system was calibrated with commercial Bap standards to generate a standard curve. Then, 500 mL of water samples were extracted with dichloromethane, and the concentrated solution was transferred to glass microvials for GC–MS analysis. Bap concentration was determined by comparison with the standard curve. Upon the exposure to 10 and 100 µg/L of Bap, their actual concentrations in water samples were determined to be 8.37 and 90.14 µg/L, respectively. Before renewing the ASW at 24 h, the amounts of Bap in the three treatment groups were 6.34 and 72.81 µg/L. Meanwhile, Bap was not detectable at either time in the control or solvent control groups.

### Histological analysis

The gills specimens were vivisected from 0 to 48 hpe clams and immediately fixed in 4% paraformaldehyde solution for 48 h, washed with 70% ethanol, then dehydrated and embedded in paraffin. The paraffin-embedded tissue was cut into 4 μm sections, followed by fixation and hematoxylin–eosin (H.E) staining according to standard procedure. After that, a slide scanning system (Pannoramic SCAN, 3DHISTECH Ltd, Budapest, Hungary) was employed to image H.E slides. Images were then viewed and analyzed with Caseviewer software (3DHISTECH Ltd, Budapest, Hungary).

### Assays of enzyme activities

To measure the activities of antioxidant and choline enzymes, fresh gills were ground into a fine homogenate using ice-cold physiological saline. Then, the mixture was centrifuged at 12,000 rpm for 15 min at 4 °C. The supernatant was immediately collected to detect the enzyme activity by a UV-spectrophotometer (Beijing Purkinje General Instrument Co., Ltd.) at wavelength of 550 nm (SOD), 405 nm (CAT), 420 nm (POD), 412 nm (GST and AChE) and 324 nm (ChAT), respectively, with corresponding commercial assay kits according to the manufacturer’s protocols. Results were expressed as units per milligram protein (U/mgprot). In addition, total protein content was determined by Coomassie Brilliant Blue total protein assay kit (Nanjing Jiancheng Bioengineering Institute, Nanjing, China) from the absorbance at 595 nm to calculate the enzyme activities.

### MDA content

Malondialdehyde (MDA) content represented the lipid peroxidation level, and the thiobarbituric reactive species (TBARS) assay was employed to determine MDA content at optical density 532 nm according to the method described by Ohkawa et al.^86^.

### 8-OHdG determination

8-OHdG was measured by enzyme linked immunosorbent assay (ELISA) using a commercial ELISA kit. Samples and 1 × PBS were mixed (ratio at 1:9) and fully homogenized. Subsequently, the homogenates were centrifuged at 3000 rpm 4 °C for 20 min, and the supernatant was collected for 8-OHdG detection according to the manufacturer’s protocol. The absorbance was evaluated at 450 nm with a microplate reader (TECAN, SPARK, Männedorf, Switzerland). And the standard curve linear regression equation was calculated through ELISACalc software according to the concentration and absorbance of standards. The content of 8-OHdG was expressed as ng/ml.

### Global DNA methylation

The global methylation levels of genomic DNA (gDNA) were measured using a MethyFlash Global DNA Methylation (5-mC) ELISA Easy kit (EPIGENTEK, Farmingdale, NY, USA). Briefly, gDNA was extracted from frozen gill tissue using Tissue DNA kit (OMEGA). After purified and quantified using a Nanodrop 2000 Spectrophotometer (THERMO SCIENTIFIC, DE, USA), a total of 100 ng gDNA was bound to strip-wells that are specifically treated to have a high DNA affinity. The methylated fraction of DNA is detected using capture and detection antibodies and then quantified colorimetrically by reading the absorbance (450 nm) in the microplate reader. The percentage of methylated DNA is proportional to the OD intensity measured and calculated by the following formula. The global DNA methylation level measured at 0 h were set as 100%, and the level at other time points were expressed as the percentage of 0 h.

$$ 5 - mC\% = \frac{Sample\;OD - NC\;OD}{{Slope \times 100}} \times 100\% $$

NC: negative control; Slope (OD/1%): the most linear part of the standard curve for optimal slope calculation.

### Statistical analysis

All experiments were repeated three times, and the data are expressed as the mean ± standard deviation (SD). The comparison of multiple groups was performed by two-way analysis of variance (ANOVA) followed by Tukey’s range test using SPSS 19.0 software (Chicago, Illinois, USA). A value of *p* < 0.05 was deemed statistically significant. The correlations between each biomarker responses were evaluated by Spearman’s rank correlation analysis, which was done using the *corrplot* package^87^ in R software (version 4.0.2).
